# Highly efficient electrochemical reduction of CO_2_ to CH_4_ in an ionic liquid using a metal–organic framework cathode†
†Electronic supplementary information (ESI) available: Fig. S1–S13 contain XRD, XPS, SEM, CVs, EIS, ^1^H-NMR, mechanism diagram and device diagram; Tables S1–S5 contain EIS data, electrolysis results in this work and in literatures and Tafel data. See DOI: 10.1039/c5sc03291a


**DOI:** 10.1039/c5sc03291a

**Published:** 2015-10-02

**Authors:** Xinchen Kang, Qinggong Zhu, Xiaofu Sun, Jiayin Hu, Jianling Zhang, Zhimin Liu, Buxing Han

**Affiliations:** a Beijing National Laboratory for Molecular Sciences , Key Laboratory of Colloid and Interface and Thermodynamics , Institute of Chemistry , Chinese Academy of Sciences , Beijing 100190 , China . Email: qgzhu@iccas.ac.cn ; Email: hanbx@iccas.ac.cn

## Abstract

Highly efficient electrochemical reduction of CO_2_ to CH_4_ is of great importance, but is challenging. Herein, Zn–1,3,5-benzenetricarboxylic acid metal–organic frameworks (Zn–BTC MOFs) deposited on carbon paper (CP) were used as cathodes in electrochemical reduction of CO_2_ using ionic liquids (ILs) as the electrolytes, which was the first work on combination of a MOF electrode and an pure IL electrolyte in the electrochemical reduction of CO_2_. It was found that the efficiency of the reaction depended strongly on the morphology of the Zn-MOFs. Compared with the commonly used metal electrodes, the electrochemical reaction showed much higher selectivity to CH_4_ and current density, and the overpotentials for CH_4_ is much lower. The excellent combination of the MOF cathodes and ILs opens a way for reduction of CO_2_ to CH_4_ effectively.

## Abstract 2

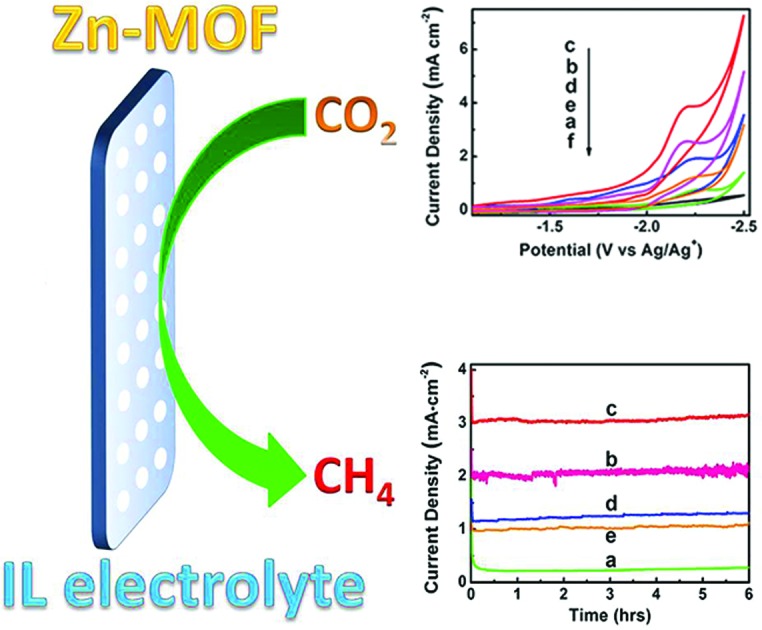
It has been discovered that Zn metal-organic framework (Zn-MOF) electrodes and ionic liquids are an excellent combination for the efficient and selective reduction of CO_2_ to CH_4_.

## Introduction

Electrocatalysis combines the advantages of efficient conversion of electrical energy into chemical energy with the convenience and stability of heterogeneous catalysis, which has received much attention.^1^ Transformation of CO_2_ into useful chemicals and fuels is very attractive because it is a cheap and renewable carbon source, but is very difficult because CO_2_ is thermodynamically stable and kinetically inert.^2^ Electrochemical reduction is a promising method in CO_2_ transformation.^3^ It can be transformed into various products, such as CO, acids, alcohols and hydrocarbons.^4^ Electrochemical reduction of CO_2_ to CH_4_ is an alternative route for synthesizing energy-rich and clean fuels, which however currently suffers from low activity and poor selectivity.^5^

Metal–organic frameworks (MOFs) represent a class of hybrid materials comprised of ordered networks formed *via* combining metal ions with organic ligands.^6^ MOFs are widely studied for gas storage and capture,^7^ separation,^8^ drug delivery^9^ and catalysis.^10^ In addition, MOFs have been used as efficient electrodes in fuel cell systems^11^ and reduction of CO_2_ in aqueous or organic electrolytes.^12^

Ionic liquids (ILs) have attracted considerable attention owing to their unique properties, such as low melting point, negligible vapor pressure, high ionic conductivity, high chemical stability, and adjustable physical and chemical properties.^13^ Applications of ILs in different fields have been studied extensively,^14^ including those in material synthesis^15^ and electrochemistry as electrolytes.^16^

The electrodes and electrolytes are crucial in electrocatalysis, and different electrodes and electrolytes can induce different products. Exploring innovative combination of catalysts and electrolytes is a very interesting topic of great importance. Herein, we conducted the first work on the electrochemical reduction of CO_2_ in MOF electrode/IL electrolyte system. It was found that the combination of MOF electrodes and ILs was very effective for the electrochemical reduction of CO_2_ to CH_4_, and the morphology of MOFs and the properties of the ILs affected the current density and selectivity to CH_4_ significantly.

## Results and discussion

**Fig. 1:**
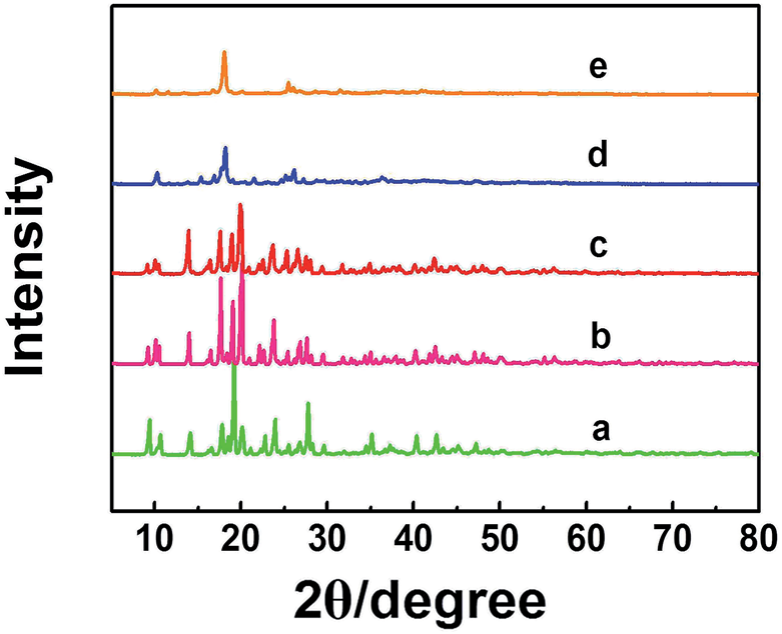
XRD patterns of the Zn-MOFs synthesized at *x* = 0.17 (a), 0.29 (b), 0.38 (c), 0.44 (d) and 0.50 (e).

Zn-MOFs can be synthesized easily by coordination of Zn^2+^ and 1,3,5-benzenetricarboxylic acid (H_3_BTC) in solution.^17^ In this work, we prepared the Zn-MOFs in the mixed solvent consisting of 75 wt% 1-dodecyl-3-methylimidazolium chloride (C_12_mimCl) and 25 wt% glycerol. The mass fractions of ZnCl_2_ (*x*) in the C_12_mimCl + glycerol + ZnCl_2_ system were 0.17, 0.29, 0.38, 0.44, 0.50, respectively. The powder X-ray diffraction (XRD) patterns of the Zn-MOFs are illustrated in Fig. 1. The results indicate that the XRD patterns of the MOFs synthesized at *x* = 0.17, 0.29, 0.38 are similar to that of the reported Zn-MOF.^18^ However, at the larger *x* values, the Zn-MOFs showed lower crystallinity (Fig. 1). The scanning electron microscopy (SEM) images of the Zn-MOFs are shown in Fig. S1.† The Zn-MOFs had the rod-like morphology at smaller *x* value, and became shorter and thicker with increasing ZnCl_2_ mass fraction. When the mass fraction of ZnCl_2_ reached 0.38, the Zn-MOFs had sheet-like morphology. The Zn-MOFs were spherical with further increasing the mass fraction of ZnCl_2_. It is well known that there existed ordered aggregates in ILs systems.^15^ In this work, small angle X-ray scattering (SAXS) technique was used to study the microstructures of the synthetic media.15*c* The results indicated that the domains in the solution varied from rod-like to sheet-like and to spherical with the increase of *x* values (Fig. S1†). This demonstrates that the morphologies of the Zn-MOFs are similar to that of the domains in the solutions in which they were formed. The detailed discussion on the Zn-MOF formation and shape control are given in the ESI† in combination with SAXS study (Fig. S1–S3† and the related discussion).

**Fig. 2:**
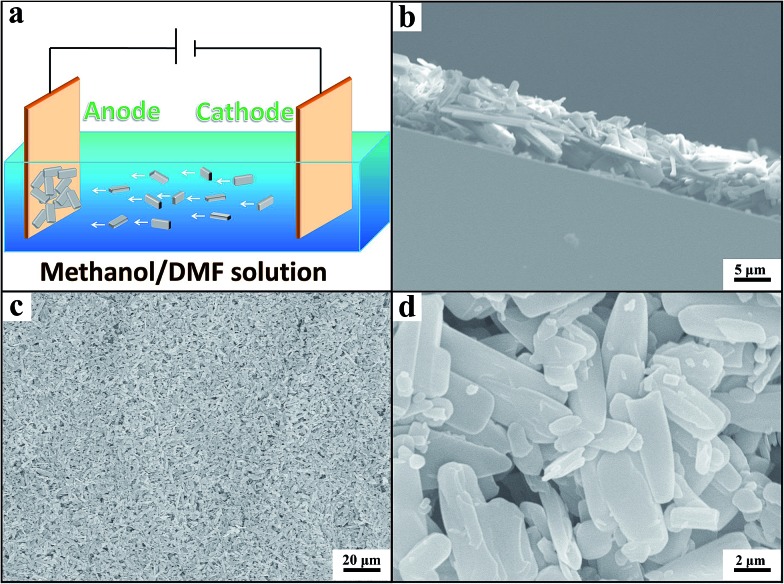
The schematic diagram of fabrication procedure and SEM images of Zn-MOF/CP cathode using the Zn-MOF synthesized at *x* = 0.38. (a) Schematic diagram of fabrication procedure. (b) SEM image to show the thickness Zn-MOF on the CP. (c) SEM image of the surface. (d) Amplified SEM image of the surface.

**Fig. 3:**
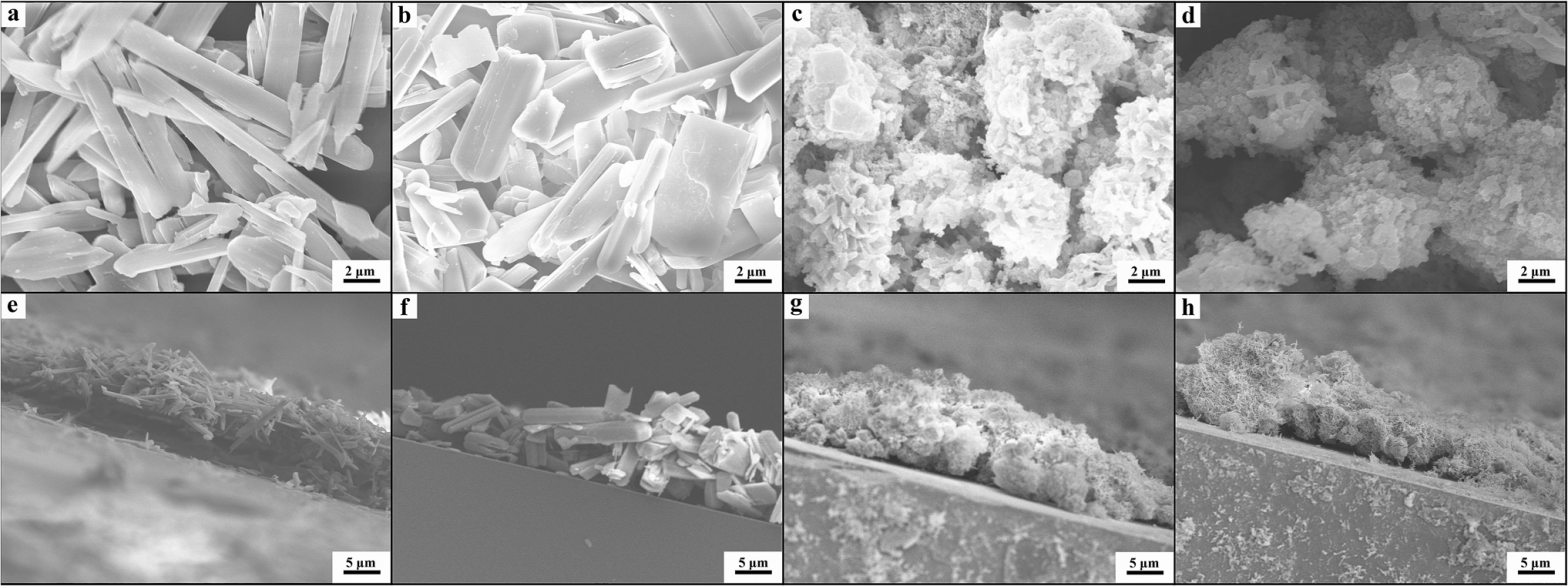
The SEM images of the morphologies of Zn-MOF/CP cathodes (a–d) and the thickness of the Zn-MOF (e–h) fabricated by EPD method. The Zn-MOF/CP cathodes were prepared using Zn-MOFs synthesized at *x* = 0.17 (a and e), 0.29 (b and f), 0.44 (c and g) and 0.5 (d and h).

We prepared the Zn-MOF/CP cathodes by depositing Zn-MOFs on the CP using the electrophoretic deposition (EPD) method.^19^ The principle of the EPD method is shown schematically in Fig. 2a and the procedures are discussed in detail in the ESI.† The Zn-MOFs synthesized can be easily deposited on CP cathode with lower voltage and short time using the electrophoretic deposition (EPD) method, the main reason is that the Zn-MOFs have partial charge on the surface due to the synthetic media.^19^ From the SEM images (Fig. 2b–d), it can be clearly seen that the Zn-MOF/CP cathode prepared using the Zn-MOF synthesized at *x* = 0.38 had smooth surface with a thickness of about 10 μm. The Zn-MOF before and after EPD were characterized by X-ray photoelectron spectroscopy (XPS) (Fig. S4†). The two peaks at 1021.9 eV and 1045.1 eV, which are assigned to the 2p_3/2_ and 2p_1/2_ components, respectively, were not changed in the EPD process, indicating that the Zn-MOF was stable in the process. The SEM images of the surface and thickness of Zn-MOF/CP cathodes prepared using the Zn-MOFs synthesized at different *x* values are shown in Fig. 3. The thicknesses of the Zn-MOFs on cathodes were also about 10 μm, however the cathodes prepared using Zn-MOFs synthesized at *x* = 0.44 and 0.5 had irregular surface. The morphologies of Zn-MOF/CP cathodes after EPD process (Fig. 2 and 3) were similar with the as-synthesized Zn-MOFs (Fig. S1†), indicating the EPD process can not destroy the morphology of Zn-MOFs. The electrochemical surface areas of the Zn-MOF/CP cathodes prepared using Zn-MOFs synthesized at *x* = 0.17, 0.29, 0.38, 0.44 and 0.50 were examined by studying the redox reactions using cyclic voltammetry (CV) shown in Fig. S5,† which were 0.40 cm^2^, 0.47 cm^2^, 1.34 cm^2^, 1.05 cm^2^ and 0.67 cm^2^, respectively. The Zn-MOF/CP cathode prepared using the Zn-MOF synthesized at *x* = 0.38 had largest electrochemical surface area because mainly of its sheet-like structure.

**Fig. 4:**
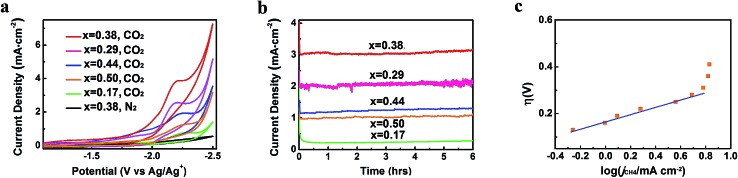
CO_2_ reduction performance of Zn-MOF/CP cathodes. (a) CV traces. (b) Current density profiles. (c) Tafel plot for CH_4_ of the Zn-MOF/CP cathode using Zn-MOF synthesized at *x* = 0.38.

CO_2_ reduction activities of different Zn-MOF/CP cathodes were investigated in CO_2_-saturated and N_2_-saturated IL 1-butyl-3-methylimidazolium tetrafluoroborate (BmimBF_4_), and the CV curves are shown in Fig. 4a. The reduction peak at about –2.2 V *vs.* Ag/Ag^+^ can be observed for the CO_2_-saturated system, while current density of the N_2_-saturated system was negligible, indicating the reduction of CO_2_. The results in Fig. 4a also demonstrate that the morphology of the Zn-MOFs affected the current density significantly. The sheet-like Zn-MOF synthesized at *x* = 0.38 showed highest current density. To explore the kinetic effect of Zn-MOFs, the electrochemical impedance spectroscopy (EIS) was conducted to study the features of the Zn-MOF/CP electrodes in BmimBF_4_, and the detailed discussion are provided in the ESI (Fig. S6–S8 and Tables S1 and S2†). The EIS result confirms that charge transfer can easily occur on the Zn-MOFs surface. The Zn-MOF/CP cathode prepared using Zn-MOF synthesized at *x* = 0.38 had the lowest charge transfer resistance (*R*_ct_) value due to the sheet-like structure with highest electrochemical surface area as discussed above.

In order to confirm the electrocatalytic response shown in Fig. 4a, the controlled potential electrolysis (CPE) experiments were carried out. The electrolysis device is shown schematically in Fig. S9.† The current densities using different Zn-MOF/CP cathodes are exhibited in Fig. 4b, which shows that the current densities did not decrease with time in the electrolysis, suggesting that the Zn-MOF electrode and the IL were stable. The CV and CPE using CP as cathode were also studied (Fig. S10†). The CP cathode produced much lower current density than Zn-MOF/CP cathode. The Zn-MOF/CP cathodes prepared from the sheet-like Zn-MOF synthesized at *x* = 0.38 generated highest current density due to its largest electroactive surface area. Therefore, CO_2_ reduction using this cathode was further studied, and the results are discussed in the following.

**Fig. 5:**
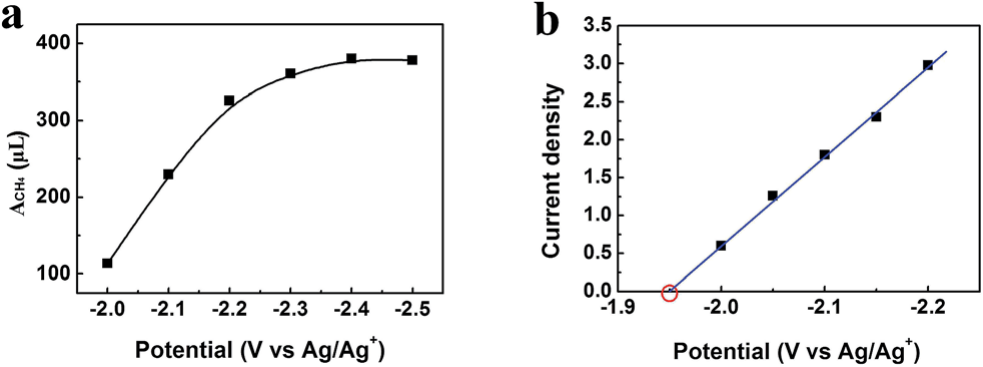
(a) Amount of CH_4_ (*A*_CH_4__, volume at standard temperature and pressure) generated in 2 h under different potentials. (b) Current densities for CH_4_ under different potentials and the equilibrium potential can be obtained by extrapolation method.

After electrolysis of 2 h at –2.2 V *vs.* Ag/Ag^+^, the gaseous product in the headspace was collected and analyzed by gas chromatography (GC), and the liquid mixture was analyzed by ^1^H-NMR to quantify liquid products (Fig. S11†). There was no product found in the liquid phase. CH_4_ was the dominate product in the gas phase with small amount of CO and H_2_. High selectivity to CH_4_ is very difficult to realize using conventional electrolysis systems. We also conducted the electrolysis under different potentials, and the amount of CH_4_ (*A*_CH_4__) is shown in Fig. 5a. It can be clearly seen that CH_4_ production rate increased dramatically at the potentials less negative than –2.2 V *vs.* Ag/Ag^+^, and rose very slowly at the potentials more negative than –2.2 V *vs.* Ag/Ag^+^. Therefore, electrolysis under –2.2 V *vs.* Ag/Ag^+^ was most suitable for CH_4_ production. CH_4_ began to generate at –1.95 V from extrapolation method using the current densities for CH_4_ under different potentials (Fig. 5b). The distinct pre-feature (Fig. 4a) at the potential less negative than –1.95 V *vs.* Ag/Ag^+^ was originated mainly from the generation of CO. The overpotential for CH_4_ was 0.25 V for this process at –2.2 V *vs.* Ag/Ag^+^ with the current density of 3.1 mA cm^–2^. Moreover, the data from Tafel plot (Fig. 4c), which was obtained by electrolysis voltage, was linear in the range of *η* = 0.19–0.37 V with the Tafel slope of 146 mV decade^–1^, indicating a rate-determining initial electron transfer to CO_2_ to form an adsorbed CO_2_˙^–^ intermediate.^20^ XPS spectra of the Zn-MOF were given before and after the electrolysis in Fig. S4.† The results demonstrated that the spectra did not change notably, further indicating the excellent stability of the Zn-MOF/CP cathode. In addition, the electrolyte was also stable according to the ^1^H-NMR spectra (Fig. S11†).

**Fig. 6:**
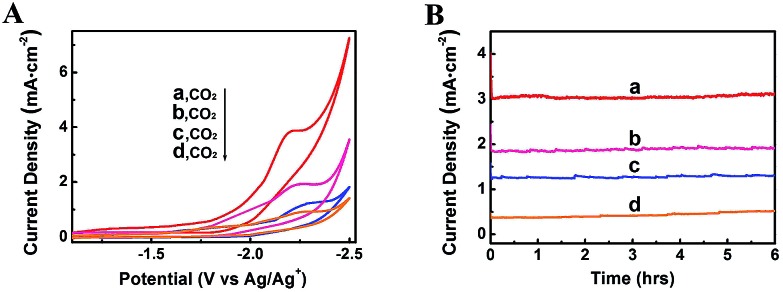
CV traces (A) and current density profiles at an applied potential of –2.2 V *vs.* Ag/Ag^+^ (B) using different kinds of ILs as electrolytes: (a) BmimBF_4_; (b) BmimOTf; (c) BmimPF_6_; (d) BmimClO_4_.

The property of electrolytes often influences an electrochemical process significantly. It was reported that imidazolium based ILs can interact with CO_2_ by physical absortption,^21^ and the ILs can serve as both robust electrolytes and CO_2_ activation promoters.^22^ In addition, they have wide electrochemical windows and good conductivity.^23^ Therefore, in this work, some other typical imidazolium based ILs were also used as the electrolytes in the electrolysis to reduce CO_2_, including 1-butyl-3-methylimidazolium trifluoromethanesulfonate (BmimOTf), 1-butyl-3-methylimidazolium hexafluorophosphate (BmimPF_6_) and 1-butyl-3-methylimidazolium perchlorate (BmimClO_4_). The CV traces and current density profiles are shown in Fig. 6, and the total current densities and faradaic efficiencies for CH_4_, CO and H_2_ are listed in Table 1. The ILs containing fluorine such as BmimBF_4_, BmimPF_6_ and BmimOTf exhibited much higher *j*_tot_ than the ILs without fluorine, which is partially because fluorine has strong interaction with CO_2_.^24^ The viscosities of the ILs also affected the *j*_tot_, as can be known from Table 1. We also conducted the electrolysis using the Zn-MOF electrode combined with other electrolytes, including DMF containing 0.01 M tetrabutylammonium tetrafluoroborate (TBABF_4_), MeCN containing 0.1 M tetrabutylammonium hexafluorophosphate (TBAPF_6_), and MeCN containing 0.1 M BmimBF_4_, and results are listed in Table S3.† The results show that the faradaic efficiency for CH_4_ was very low when these electrolytes were used, indicating that imidazolium based ILs were crucial for the very high selectivity to CH_4_.

**Table 1:** CO_2_ reduction performance of Zn-MOF/CP cathodes in imidazolium based IL electrolytes after 2 h at an applied potential of –2.2 V *vs.* Ag/Ag^+^^*a*^

Entry	Electrolytes	*η* ^*b*^ [cp]	*j*_tot_ [mA cm^–2^]	FE_CH_4__ [%]	FE_CO_ [%]	FE_H_2__ [%]
1	BmimBF_4_	140	3.1 ± 0.5	80.1 ± 6.6	7.9 ± 2.6	12.0 ± 3.3
2	BmimOTf	93	2.1 ± 0.3	85.4 ± 3.2	4.6 ± 1.2	10.0 ± 2.5
3	BmimPF_6_	366	1.6 ± 0.3	87.7 ± 5.1	5.4 ± 2.0	6.9 ± 3.0
4	BmimClO_4_	48	0.5 ± 0.2	88.3 ± 3.8	6.8 ± 2.1	4.9 ± 1.0

^*a*^The Zn-MOF/CP cathode was prepared using Zn-MOF synthesized at *x* = 0.38 for reduction of CO_2_.

^*b*^Viscosity of ILs at 25 °C. The data was obtained from the Centre of Green Chemistry and Catalysis, LICP, CAS.

**Fig. 7:**
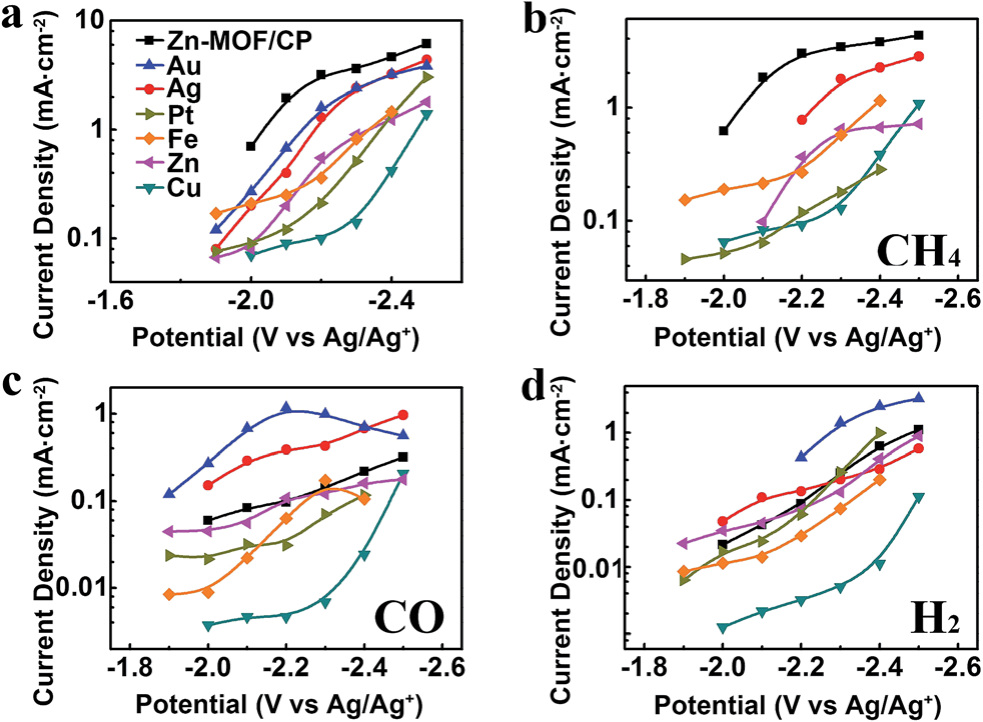
Current densities obtained from of the Zn-MOF/CP cathode prepared using Zn-MOF synthesized at *x* = 0.38 and various metal cathodes. (a) Total current densities. (b) Partial current densities of CH_4_. (c) Partial current densities of CO. (d) Partial current densities of H_2_.

Metal electrodes are commonly used to reduce CO_2_. In this work, we carried out the electrolysis using Au, Ag, Pt, Fe, Zn, and Cu cathodes in BmimBF_4_ at different voltages as well. The metal electrodes used were polished metal foils with bulk structure, therefore the effect of the surface structure can be ignored. The *j*_tot_ and partial current densities of CH_4_ (*j*_CH_4__), CO (*j*_CO_) and H_2_ (*j*_H_2__) over the potential range from –1.9 V to –2.5 V *vs.* Ag/Ag^+^ are compared with the results obtained from the Zn-MOF cathode in Fig. 7. The CVs of different metal cathodes are shown in Fig. S12,† and the principal product, linear range in Tafel plot and Tafel slope for their main product are provided in Table S4.† Obviously, the *j*_tot_ and the selectivity to CH_4_ of the Zn-MOF/CP system were much larger than that of the metal cathode systems at the same voltage, suggesting that the Zn-MOF electrode was also very important for the high efficiency in producing CH_4_. The potential was less negative using Zn-MOF/CP cathode than metal cathodes to reach the same current density of CH_4_, indicating that the Zn-MOF/CP cathode was more active for CH_4_ generation. For comparison, Table S5† lists the CH_4_ selectivity reported in the literature for the electrochemical reduction of CO_2_. The data indicate that the selectivity to CH_4_ in our work was the highest. The product contained 85% CO and 15% H_2_ when CP was used as the cathode in BmimBF_4_ at –2.2 V *vs.* Ag/Ag^+^, further indicating that the Zn-MOFs played a key role for producing CH_4_. These results demonstrate that the Zn-MOF/CP cathode and the IL BmimBF_4_ are excellent combination for producing CH_4_ from electrochemical reduction of CO_2_.

As discussed above, both Zn-MOF cathodes and imidazolium based IL electrolytes are crucial for the high yield of CH_4_. The Zn-MOF/CP cathodes and imidazolium based IL electrolytes are excellent combination for producing CH_4_. In addition, the current density reached the highest value using Zn-MOF/CP cathode prepared using Zn-MOF synthesized at *x* = 0.38 in BmimBF_4_.

**Fig. 8:**
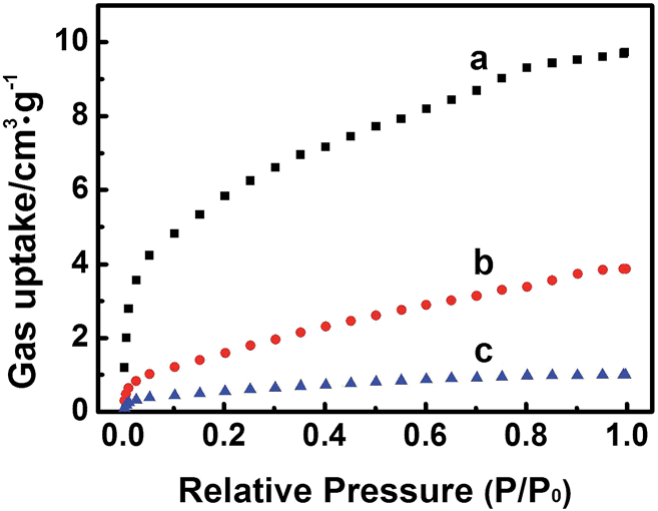
The adsorption curves of CO_2_ (a), CO (b) and CH_4_ (c) at 298 K on the Zn-MOF synthesized at *x* = 0.38.

The high electrochemical activity of the Zn-MOFs in BmimBF_4_ results partially from the facts that the imidazolium based ILs containing fluorine can absorb and activate CO_2_,^24^ and the Zn-MOFs are porous materials, which benefits gas adsorption.^7^ The Zn-MOFs were synthesized in imidazolium based IL mixture, which therefore has very good compatibility with ILs, which helps driving the reaction.^16^ In addition, it is reported that Zn-MOFs are efficient selective adsorbent for different gases.^17^ At present, there is no method to determine the gas adsorption amount on solid surface in the presence of a liquid, so we determined the adsorption amounts of the gases in the absence of IL in order to get some indirect evidence to discuss the interactions between the gases and the electrode, and the adsorption properties of the Zn-MOF for CO_2_, CO, CH_4_ at 298 K (Fig. 8) were studied. The adsorption amounts of CO_2_, CO, and CH_4_ on the Zn-MOF at 1 atm and 298 K are 9.7 cm^3^ g^–1^, 3.9 cm^3^ g^–1^, and 1.0 cm^3^ g^–1^, respectively, indicating that Zn-MOF had much stronger adsorption for CO_2_ and CO than for CH_4_. Furthermore, only CO can be detected at the potential less negative than –1.95 V *vs.* Ag/Ag^+^, and CH_4_ began to generate at –1.95 V and became dominate product soon (Fig. 5b), suggesting that CH_4_ was derived from CO. CO_2_ was absorbed on the surface of Zn-MOFs and was reduced to CO. Most of the CO molecules generated tended to be further reduced to CH_4_ because the interaction between Zn-MOF and CO is stronger than that between Zn-MOF and CH_4_.

**Fig. 9:**
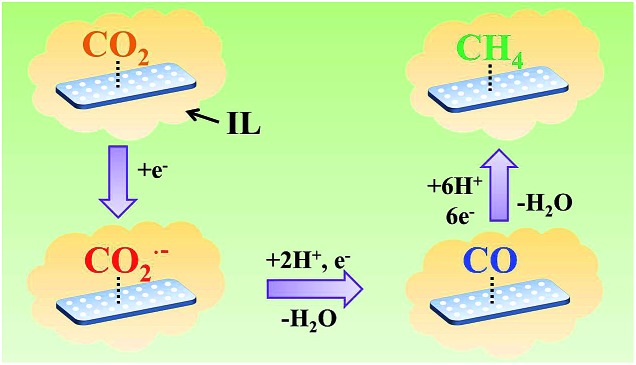
The possible pathway for electrochemical reduction of CO_2_ to CH_4_ on Zn-MOF/CP cathode in ILs.

On the basis of the results of this work and the knowledge in the literature, the possible pathway for electrochemical reduction of CO_2_ to CH_4_ using Zn-MOF/CP cathodes in imidazolium based ILs can be discussed briefly, which is shown schematically in Fig. 9. In the electrolysis, some imidazolium cations were adsorbed on the Zn-MOF surface after the Zn-MOF/CP cathode immersed in the IL electrolyte. Thus, CO_2_ molecules were captured by the IL adsorbed on the Zn-MOF surface. One electron was transferred to a CO_2_ molecule and forms CO_2_˙^–^ intermediate, and then the CO_2_˙^–^ intermediate took another electron and yields a CO molecule. In this step, the conductive Zn-MOF transferred electron to CO_2_, and imidazolium base ILs helped driving the transformation of CO_2_ to CO_2_˙^–^ intermediate. CO could be desorbed from the surface of Zn-MOF or be further reduced by six electrons to generate CH_4_.4*d* Due to the larger adsorption capacity of CO than CH_4_ on the Zn-MOF as discussed above, the CO molecule preferred to be adsorbed on the Zn-MOF surface to be further reduced to CH_4_. More detailed mechanism is very interesting, but is challenging.

In summary, the Zn-MOF/CP cathodes and IL electrolytes have been combined for electrochemical reduction of CO_2_ to CH_4_. The morphology of the Zn-MOFs has significant effect on the electrochemical reaction. The sheet-like Zn-MOF has the highest activity in the CO_2_ reduction due to its largest electroactive surface areas, and the imidazolium based ILs with fluorine are more effective electrolytes because fluorine has stronger interaction with CO_2_. The Zn-MOF based cathodes and the ILs are excellent combination for the efficient and selective reduction of CO_2_ to CH_4_. The selectivity of CH_4_ can be higher than 80% at a current density of higher than 3 mA cm^–2^ with an overpotential of 0.25 V. We believe that integration of MOF-based electrodes and ILs provides many opportunities for exploring efficient electrochemical reactions.

## Experimental section

### Materials

ILs, C_12_mimCl (purity > 99%), BmimBF_4_ (purity > 99%), BmimOTf (purity > 99%), BmimPF_6_ (purity > 99%) and BmimClO_4_ (purity > 99%) were purchased from the Centre of Green Chemistry and Catalysis, LICP, CAS. Organic salts TBABF_4_ (purity > 99%) and TBAPF_6_ (purity > 99%) were also obtained from this company. ZnCl_2_ (A. R. grade), KCl (A. R. grade), K_3_Fe(CN)_6_ (A. R. grade), glycerol (A. R. grade), acetonitrile (A. R. grade), methanol (A. R. grade), DMF (A. R. grade), metal foils (Au, Ag, Pt, Fe, Zn and Cu, purity > 99.99%) were provided by Sinopharm Chemical Reagent Co., Ltd, P. R. China. 1,3,5-Benzenetricarboxylic acid (H_3_BTC, purity > 99%) were obtained from J & K Scientific Ltd. Toray Carbon Paper (CP, TGP-H-60, 19 × 19 cm) and Nafion N-117 membrane (0.180 mm thick, ≥0.90 meg g^–1^ exchange capacity) were purchased from Alfa Aesar China Co., Ltd. Before used, the ILs were dried in vacuum oven at 80 °C for 48 h and the water content was less than 0.1 wt% as determined by Karl-Fischer method.^25^

### Zn-MOFs synthesis

The procedures to synthesize the Zn-MOFs were similar to that reported previously.^26^ In a typical experiment, 15 g C_12_mimCl, 5 g glycerol, desired amount of ZnCl_2_ and 0.8 g H_3_BTC were added into a two-necked round-bottomed flask, and the mixture was stirred vigorously at 80 °C for 72 h. After the reaction, the obtained mixture containing the materials was mixed with 50 mL ethanol and then centrifuged with a speed of 5000 rpm. The obtained Zn-MOFs were washed with ethanol for 10 times and dried in a vacuum oven at 60 °C for 24 h.

### SAXS study

SAXS experiments were carried out at Beamline 1W2A at the Beijing Synchrotron Radiation Facility. The apparatus and the procedures were similar to that used in previous work.15*c* The data were collected using a CCD detector (MAR) with maximum resolution of 3450 × 3450 pixels. The wavelength of the X-ray was 1.54 Å, and the distance of the sample to detector was 1.31 m. In a typical experiment, the sample was added into the sample cell, and the X-ray scattering data were recorded. The 2-D SAXS images were obtained from the detector and then transformed into the profiles of intensity (*I*) *vs.* wavevector (*q*) by the software FiT2D. The pair-distance distribution function *p*(*r*) was obtained from SAXS data by using Gnom application software.^26^

### Materials characterization

XRD analysis of the samples was performed on the X-ray diffractometer (Model D/MAX2500, Rigaka) with Cu-Kα radiation, and the scan speed was 2° min^–1^. The surface morphologies of the products were characterized by a HITACHI S-4800 SEM. The surface components were characterized by XPS performed on the Thermo Scientific ESCALab 250Xi using 200 W monochromated Al Kα radiation, in which the 500 μm X-ray spot was used. The adsorption isotherms of CO_2_, CO and CH_4_ of the degassed Zn-MOFs were determined at 298 K in the pressure range of 0–1 atm on a TriStar II 3020 device.

### Fabrication of Zn-MOF/CP cathodes and characterization

The EPD procedure was performed on DC power supply LW6020KD (Longwei Instrument (HK) Co. Ltd). Prior to experiments, the carbon paper (CP) substrate was sonicated in acetone for 10 min, followed by washing with water and ethanol, and finally dried in N_2_ atmosphere. The procedures were similar to that reported.^19^ For each series of EPD experiments, 1 mg Zn-MOF powder was dispersed in 10 mL methanol/DMF solution (the concentration of DMF was 20 wt%) prior to 10 min sonication. Then two CP substrates of the same size (0.5 cm × 0.5 cm) were used as anode and cathode, respectively. The Zn-MOF was deposited onto anode by applying a suitable constant voltage (10–50 V) of 1 hour, and the distance between electrodes was 2 cm. The as-deposited Zn-MOF films were washed with ethanol for several times and dried in vacuum oven at 80 °C for 24 h. The morphologies of the as-synthesized Zn-MOF/CP cathodes were characterized by SEM.

### Cyclic voltammetry (CV) study

An electrochemical workstation (CHI 6081E, Shanghai CH Instruments Co., China) was used for all CO_2_ reduction experiments. CV measurements were carried out in a single compartment cell with three-electrode configuration, which consisted of working electrode (*e.g.* Zn-MOF/CP), a platinum gauze auxiliary electrode, and Ag/Ag^+^ (0.01 M AgNO_3_ in 0.1 M TBAP–MeCN) reference electrode. The reference electrode was stabilized in a glass tube with a Luggin capillary, which was filling with corresponding catholyte. The reference electrode calibration was carried out using the method reported in the literature.^16^ The potential difference of the Ag/Ag^+^ electrode in BmimBF_4_ and standard hydrogen electrode (SHE) is 636 mV at 25 °C. The detailed results and the discussion are given in the ESI (Fig. S13†). Before each set of experiment, the electrolyte was bubbled with CO_2_ (or N_2_) for 30 min until CO_2_-saturated solution (or N_2_-saturated solution) was formed, which was confirmed by the fact that the CV trace was not changed with gas bubbling time. CV measurements in gas-saturated electrolyte were taken between –1.1 V and –2.5 V *vs.* Ag/Ag^+^ at a sweep rate of 20 mV s^–1^. For better mixing, slight magnetic stirring was applied in the process. Prior to experiments, all the metal electrodes (0.5 cm × 0.5 cm) were polished with fine sand paper and then were sonicated in acetone for 10 min, followed by washing with water and ethanol, and finally dried in N_2_ atmosphere.

### Electrochemical surface area measurement

The active surface areas in the IL may be different with that determined in the aqueous solution. However, the acceptable characterization method in ILs has not been reported. Therefore, in this work, we made the characterization using the well-accepted method as follows.^11^ The electrochemical surface areas of the Zn-MOF/CP electrodes were determined by steady-state cyclic voltammetry (CV) in a solution of 0.01 M [Fe(CN)_6_]^3–/4–^ with 1 M KCl at a scan rate of 50 mV s^–1^. The electrochemical surface area was estimated according to the Randles–Sevcik equation.^27^

### EIS measurements

The measurement was performed using the Zn-MOF/CP electrodes as the reference.^28^ The experimental apparatus were the same as for CV measurements. The impedance spectra was recorded in IL BmimBF_4_ at an open circuit potential (OCP) with an amplitude of 5 mV of 10^–2^ to 10^5^ Hz. The formal potential of the system was also set at –2.0 V similar to the CO_2_ reduction potential at the same experimental conditions. The data obtained from the EIS measurements were fitted using the Zview software (Version 3.1, Scribner Associates, USA).

### CO_2_ reduction electrolysis and product analysis

Electrolysis was performed under room temperature (25 °C) in a commonly used H-type cell with an Ag/Ag^+^ (0.01 M AgNO_3_ in 0.1 M TBAP–MeCN) reference electrode, which was similar to that used by other researchers.^3^,^16^ The apparatus was shown schematically in Fig. S9.† The cathode and anode compartments were separated by a proton exchange membrane (Nafion 117). ILs or organic solvents and H_2_SO_4_ aqueous solution (0.5 M) were used as cathodic and anodic electrolytes, respectively. The proton source was from the electrolysis of water at the anode. Before electrolysis, CO_2_ was bubbled through the catholyte (2 mL per min) for 30 min with stirring. Potentiostatic electrochemical reduction of CO_2_ was carried out with CO_2_ bubbling (2 mL min^–1^), and the gaseous product was collected in a gas bag. After a desired electrolysis time, the gaseous product in the gas bag was collected and analyzed by gas chromatography (GC, HP 4890D), which was equipped with TCD and FID detectors using helium as the internal standard, and the liquid mixture was analyzed by ^1^H-NMR method, which recorded on a Bruker Avance III 400 HD spectrometer in DMSO-d_6_ with TMS as an internal standard. The amount of CH_4_ and faradaic efficiency of the products were calculated on the basis of GC analysis.3b,^29^

Multiple electrolysis experiments were run at each potential and the average current density was calculated to give the data. The products of CO_2_ reduction *vs.* hydrogen reduction were measured at each potential. The variation in partial current density *vs.* applied overpotential was obtained *via* stepped potential electrolysis. Partial current densities for CH_4_ production were calculated from the GC spectra every 15 minutes and averaged over 1–2 hours. The Tafel plots were constructed from these data.

## Supplementary Material

Supplementary information
