# Supplementary material for: Highly efficient electrochemical reduction of CO2 to CH4 in an ionic liquid using a metal–organic framework cathode
Source: Chem Sci. 2015 Oct 2;7(1):266–73. doi: 10.1039/c5sc03291a (PMC5952524; doi:10.1039/c5sc03291a)
Supplement: Supplementary file 1 [file SC-007-C5SC03291A-s001.pdf]

Electronic Supplementary Material (ESI) for Chemical Science.
This journal is © The Royal Society of Chemistry 2015

Supplementary Information

Highly Efficient Electrochemical Reduction of CO_2 to CH_4 in Ionic Liquid using Metal-Organic Framework Cathode

Xinchen Kang, Qinggong Zhu,* Xiaofu Sun, Jiayin Hu, Jianling Zhang, Zhimin Liu, Buxing Han*

Beijing National Laboratory for Molecular Sciences, Institute of Chemistry, Chinese Academy of Sciences, Beijing 100190, China

Contents

Results and discussion---------------------------- --2

Supplementary Figures---------------------------- --4

Supplementary Tables------------ ---12

References ------------ ---15

Results and discussion

Zn-MOF synthesis

Ionic liquids (ILs) are excellent solvents to dissolve the precursors of MOFs.[S1] It has been claimed that the phase behavior, intermolecular interaction and microstructure are the most critical aspects in the application of IL-containing systems. The microstructures of the synthetic media influence the morphology of the Zn-MOFs significantly.[S2] In this work, the solvent consisted of 75 wt% C_{12}mimCl and 25 wt% glycerol. The mass fractions of $ZnCl_2$ (x) in the C_{12}mimCl + glycerol + $ZnCl_2$ system were 0.17, 0.29, 0.38, 0.44, 0.50, respectively.

The microstructure of the C_{12}mimCl + glycerol + $ZnCl_2$ system with different $ZnCl_2$ contents were characterized by SAXS method, and the results are given in Fig. S1, together with the SEM images of the Zn-MOFs synthesized. The domains in the solution were rod-like at lower x, and the rod-like Zn-MOFs were formed. With the increase of the x, the length-width ratio L/D_c of the IL/glycerol/$ZnCl_2$ domains decreased, which resulted in podgy rods. When the x reached 0.38, the domains in the system became sheet-like, and the Zn-MOF was sheet-like material synthesized at this condition. The domains became spherical when x was higher than 0.44, which can be known because the ordered structure was completely disappeared (Fig. S2). The Zn-MOFs synthesized at the conditions had spherical morphology, which was consistent with poorer crystallinity of the Zn-MOFs prepared at this condition (Fig. 1). With x increasing continuously, the domains in the system became larger, which resulted mainly from the high viscosity of the solution, and larger Zn-MOFs spheres were formed.

The results above indicate that the shape of the Zn-MOFs is similar to that of the domains in the C_{12}mimCl + glycerol + $ZnCl_2$ system. This is easy to understand, and is discussed taking the formation of the sheet-like Zn-MOFs as example, which is shown schematically in Fig. S3. The ligands existed in the C_{12}mimCl + glycerol + $ZnCl_2$ system, and the sheet-like Zn-MOFs nuclei were formed by coordination reaction of the Zn^{2+} and BTC in the domains. The sheet-like Zn-MOF nuclei were formed because the domains acted as the templates. The nuclei behaved as seeds of the Zn-MOFs and grew gradually to form larger sheet-like particles.

Higher $ZnCl_2$ concentration in the synthesis solution resulted in higher Zn content in the as-synthesized Zn-MOFs. The diffraction peaks moved to lower 2θ degree, indicating the enlarged interplanar spacing d (Fig. 1), which illustrating that excess Zn^{2+} ions incorporated interstitially instead of segregate out of the MOFs.

Electrochemical impedance spectroscopy (EIS)

The interfacial effect between Zn-MOF/CP electrode and ionic liquid on the influence of CO_2 reduction was characterized by EIS. Fig. S6 shows the EIS of each Zn-MOF/CP electrode at an open circuit potential (OCP) and -2.0 V closer to the CO_2 reduction potential. A simple equivalent circuit (Fig. S7) is used for simulating the experimental impedance data. The resulting experimental and simulated EIS spectra

of various Zn-MOF/CP cathodes with Randles' equivalent circuit R(C(R(Q(RW)))) are illustrated in Fig. S8, and values of the main parameters of Randles equivalent circuit elements obtained by fitting the EIS spectra are listed in Supplementary Tables 1 and 2. Relevant data extracted from Table S1 represents a pure capacitive behavior of Zn-MOF/CP electrode in $BmimBF_4$,[S3] indicating the Zn-MOFs have partial charge on the surface. The capacitive behavior could also be explained the presence of a new molecule in the double layer for Zn-MOF electrode when a highly ordered ionic layer is generated on the electrode surface. Since the charge transfer resistance (R_{ct}) which is related to electronic transfer occurring at the interface will strongly depend on the double layer properties,[S4] and the R_{ct} values obtained at OCP only reflect the electronic transfer ability of the electrode. It is concluded that electrode fabricated from the sheet-like Zn-MOF synthesized at x=0.38 had lowest R_{ct} value of 16.83 $\Omega\cdot cm^2$. In contrast, the contact surface between the rod-like or between the spherical Zn-MOFs particles is small, and thus the charge transfer is blocked significantly in Zn-MOFs electrodes synthesized. On the other hand, there is a decrease in the value of R_{ct} when the formal potential was set at -2.0V closer to the onset potential of CO_2 reduction. The decreasing R_{ct} and increasing CPE values are related to de deformation of the double layer due to the inclusion of CO_2 molecules, and the migration of species to the electrode allows an easier electron transfer on the electrode surface.

Reference Electrode Calibration

The calibration was based on the reported literatures.[S5-S6] In the experiment, a ferrocene/ferrocenium (2.5mM) redox couple in acetonitrile and $BmimBF_4$ is used to calibrate the reference electrode with scan rate of 10 mV/s, which is shown in Fig. S13. We found the potential difference of the redox couple in acetonitrile and in $BmimBF_4$ was 99 mV. Therefore, we conclude that there is a 537+99=636mV difference between the potential of the designed electrode in $BmimBF_4$ and SHE at 25 ^oC, indicating that the influence of the addition resistance is negligible in IL medium.

Supplementary Figures

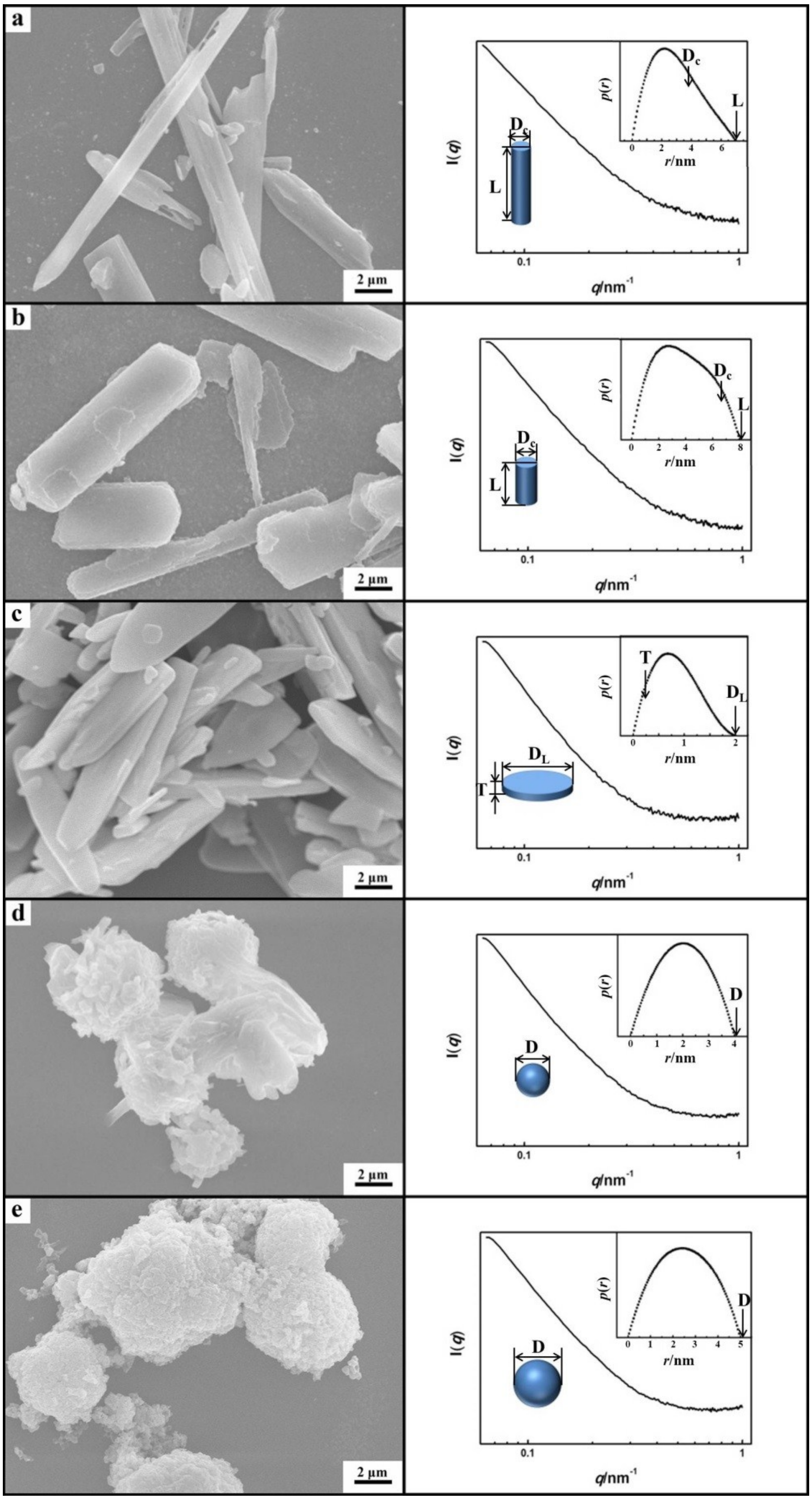

Fig. S1 SEM images of the Zn-MOFs (left) and SAXS curves (right). The solvent consisted of 75 wt% C_{12}mimCl and 25 wt% glycerol. The mass fractions of $ZnCl_2$ (x) in the C_{12}mimCl + glycerol + $ZnCl_2$ system were 0.17 (a), 0.29 (b), 0.38 (c), 0.44 (d), 0.50 (e).

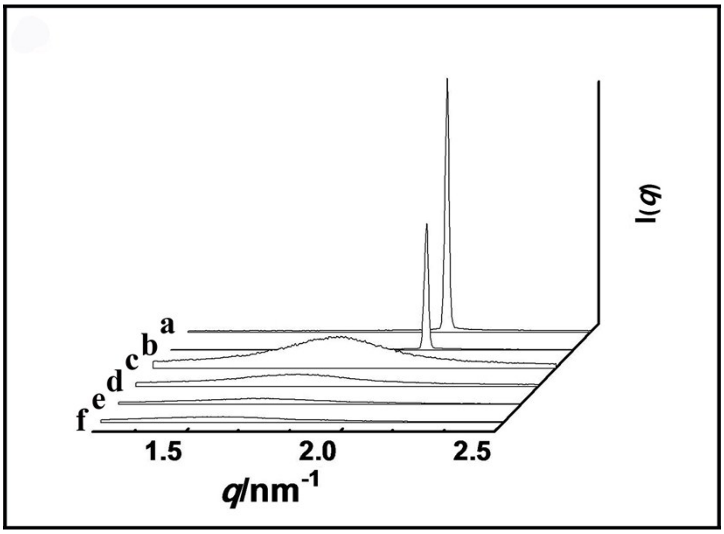

Fig. S2 The SAXS curves of C_{12}mimCl + glycerol + $ZnCl_2$ system. The solvent consisted of 75 wt% C_{12}mimCl and 25 wt% glycerol. The mass fractions of $ZnCl_2$ (x) in the C_{12}mimCl + glycerol + $ZnCl_2$ system were 0 (a), 0.17 (b), 0.29 (c), 0.38 (d), 0.44 (e) and 0.50 (f). The sharp peaks indicate the existence of the ordered structure in the mixture.

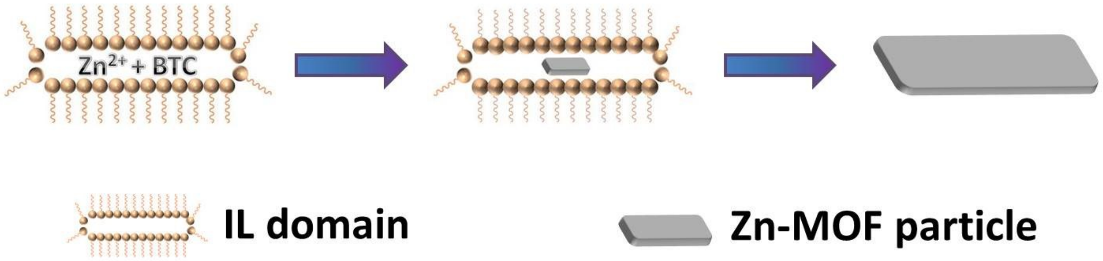

Fig. S3 Schematic diagram to show the formation mechanism of the Zn-MOF sheets.

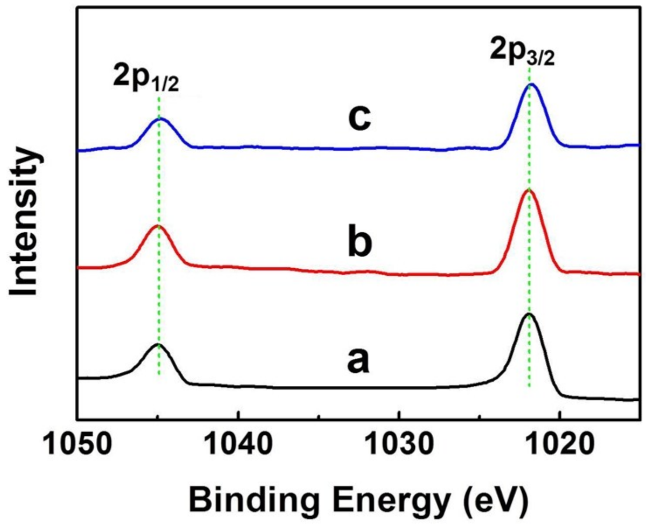

Fig. S4 Zn 2p XPS spectra of Zn-MOF (a), Zn-MOF/CP cathode (b), and Zn-MOF/CP cathode after electrolysis (c). The Zn-MOF cathode was prepared using Zn-MOF synthesized at x=0.38.

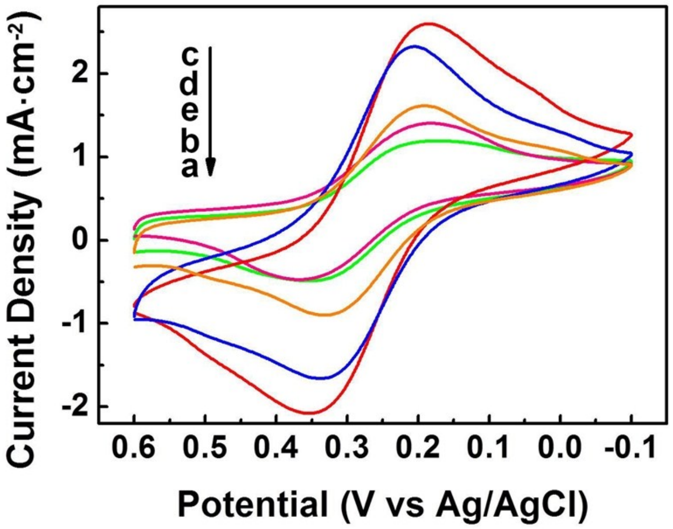

Fig. S5 CVs of 10 mM $Fe(CN)_6^{3-/4-}$ in 1 M KCl using different Zn-MOF/CP cathodes prepared using Zn-MOFs synthesized at x=0.17 (a), 0.29 (b), 0.38 (c) 0.44 (d) and 0.5 (e).

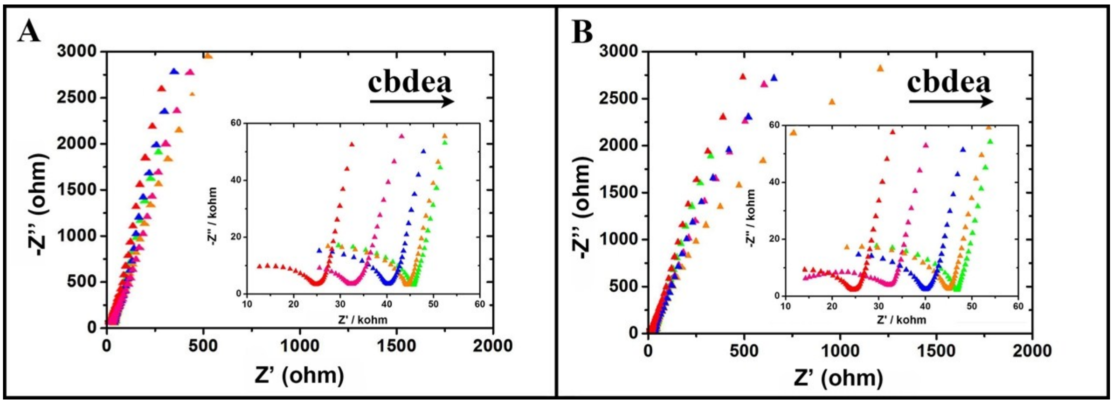

Fig. S6 Nyquist plots for various Zn-MOF/CP cathodes prepared using Zn-MOFs synthesized at x=0.17 (a), 0.29 (b), 0.38 (c), 0.44 (d), 0.50 (e) in $BmimBF_4$ at OCP and -2.0 V under CO_2 atmospheres.

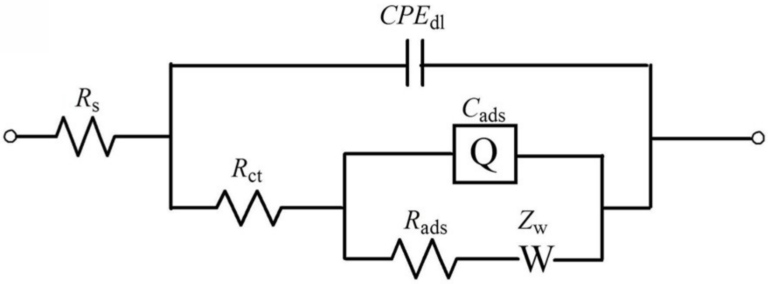

Fig. S7 Electrical equivalent circuit used for simulating the experimental impedance data. The components contain solution resistance (R_s), electron transfer resistance (R_{ct}), double layer capacitance (CPE_{dl}), surface adsorption capacitance (C_{ads}), surface adsorption resistance (R_{ads}) and Warburg-type impedance (Z_w).

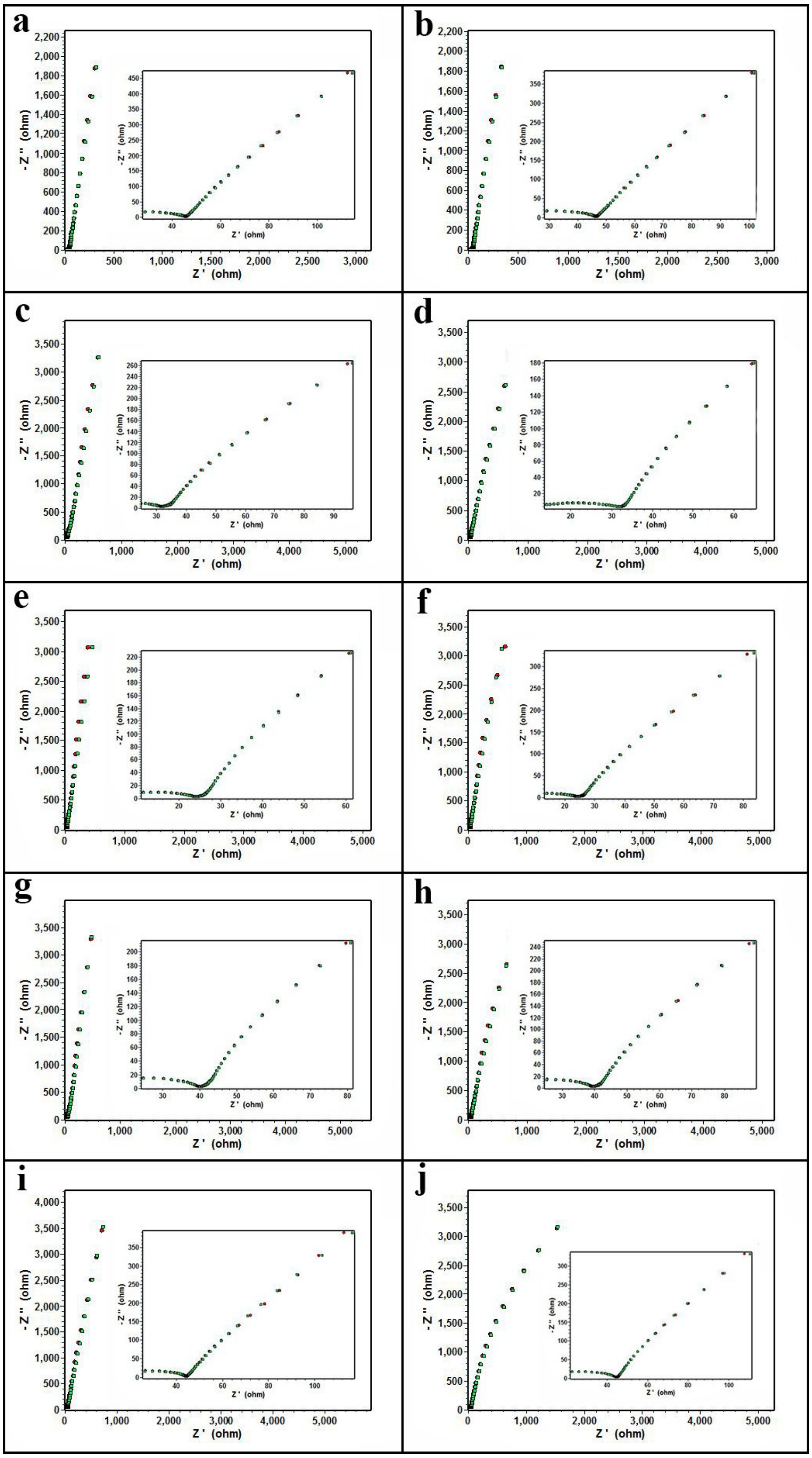

Fig. S8 The experimental and simulated EIS spectra of Zn-MOF/CP cathodes prepared using Zn-MOFs synthesized at x=0.17 (a-b), 0.29 (c-d), 0.38 (e-f), 0.44 (g-h), 0.50 (i-j) with Randles' equivalent circuit R(C(R(Q(RW)))) at OCP (left column) and -2.0 V (right column).

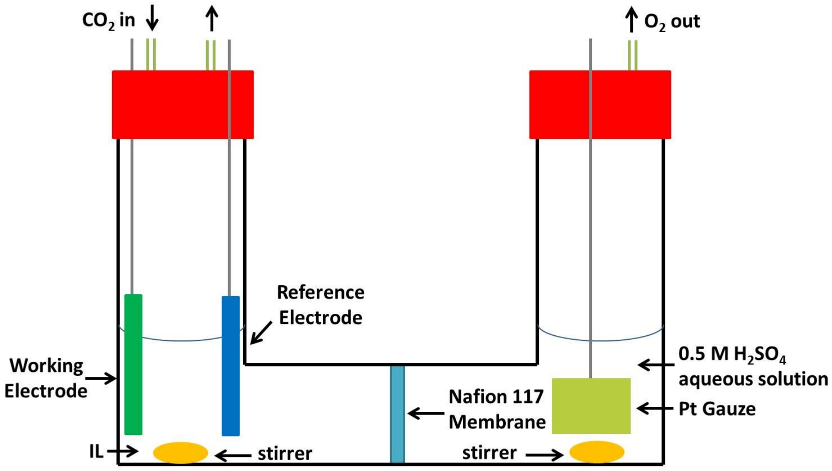

Fig. S9 The schematic diagram of the electrolysis device.

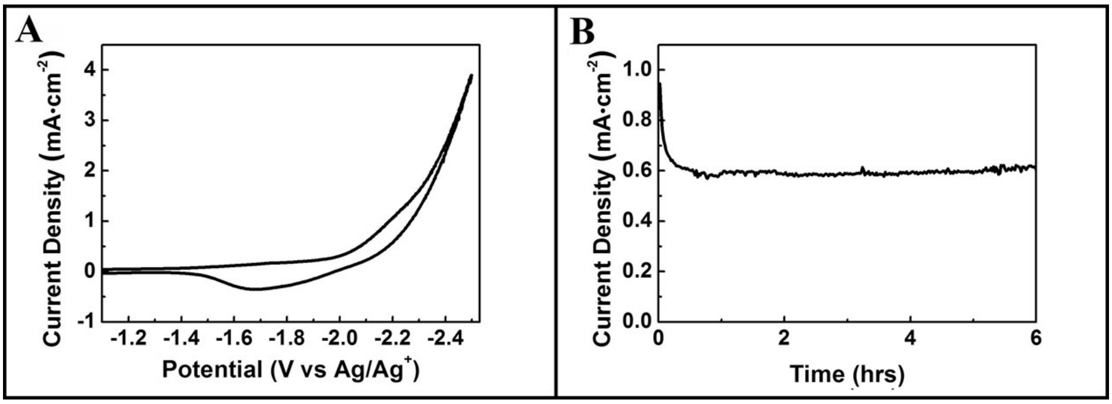

Fig. S10 CV and Current density profile using CP as cathode.

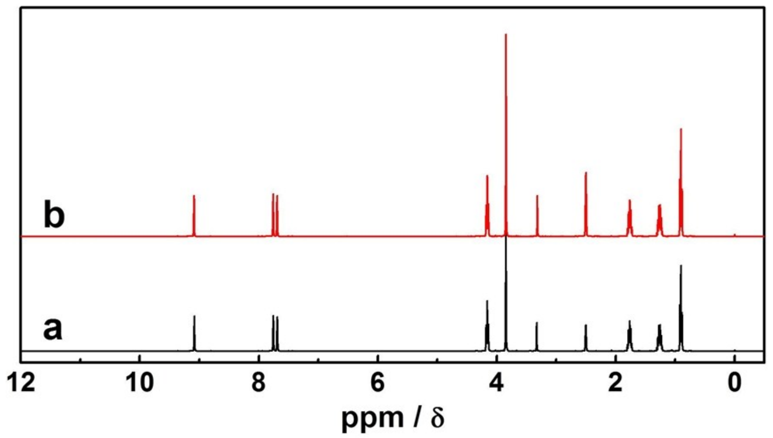

Fig. S11 ^{1}H-NMR spectra of the electrolyte $BmimBF_4$ before (a) and after (b) electrolysis in DMSO-d_6 with TMS as an internal standard.

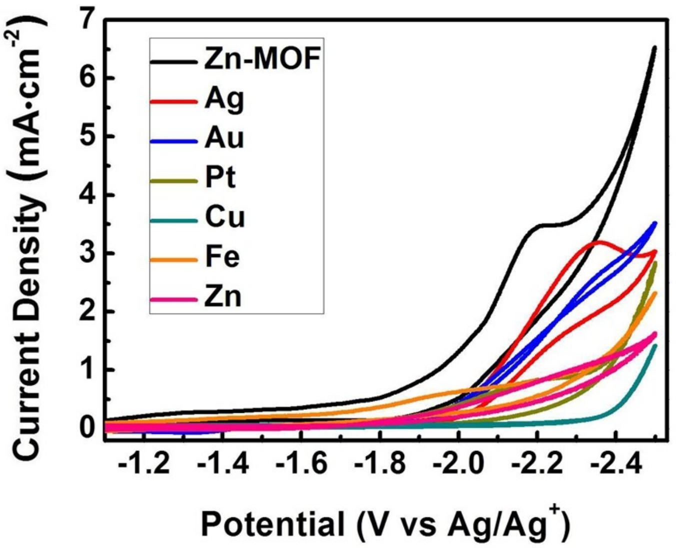

Fig. S12 CV traces obtained in $BmimBF_4$ using different kinds of electrodes. The Zn-MOF/CP cathode was prepared using Zn-MOF synthesized at x=0.38.

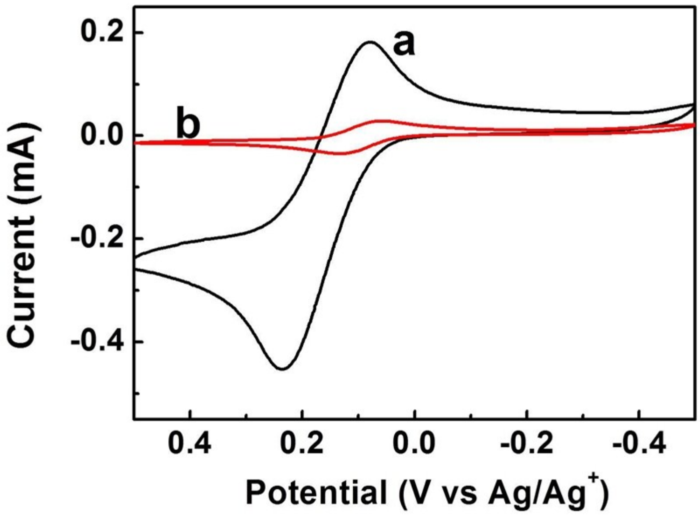

Fig. S13 Anodic and cathodic wave for ferrocene/ferrocenium (2.5mM) redox couple in Acetonitrile (a) and $BmimBF_4$ (b) using the Ag/Ag^+ reference.

Supplementary Tables

Table S1 Values of the main parameters of Randles equivalent circuit elements obtained by fitting the EIS spectra with Randles' equivalent circuit R(C(R(Q(RW)))) at OCP.

Entry	x[a]	R_s ($\Omega \cdot cm^2$)[b]	CPE ($\Omega^{-1} \cdot cm^{-2} \cdot S^n$)[c]	n[d]	R_{ct} ($\Omega \cdot cm^2$)[e]
1	0.17	11.37	9.3×10^{-5}	0.80	34.31
2	0.29	10.61	8.4×10^{-5}	0.87	19.28
3	0.38	5.39	10.2×10^{-5}	0.80	16.83
4	0.44	10.34	10.8×10^{-5}	0.80	29.93
5	0.50	15.71	11.7×10^{-5}	0.91	33.68

[a]The mass fraction of $ZnCl_2$ (x) in the C_{12}mimCl + glycerol + $ZnCl_2$ systems. [b]R_s is solution resistance. [c]CPE is double layer capacitance. [d]n is dimensionless parameter. [e]R_{ct} is electron transfer resistance.

Table S2 Values of the main parameters of Randles equivalent circuit elements obtained by fitting the EIS spectra with Randles' equivalent circuit R(C(R(Q(RW)))) at -2.0 V.

Entry	x[a]	R_s ($\Omega \cdot cm^2$)[b]	CPE ($\Omega^{-1} \cdot cm^{-2} \cdot S^n$)[c]	n[d]	R_{ct} ($\Omega \cdot cm^2$)[e]
1	0.17	11.16	9.9×10^{-5}	0.91	33.2
2	0.29	12.57	11.5×10^{-5}	0.89	19.16
3	0.38	5.98	11.8×10^{-5}	0.89	16.10
4	0.44	10.03	12.2×10^{-5}	0.90	18.69
5	0.50	10.87	13.0×10^{-5}	0.87	19.86

[a]The mass fraction of $ZnCl_2$ (x) in the C_{12}mimCl + glycerol + $ZnCl_2$ systems. [b]R_s is solution resistance. [c]CPE is double layer capacitance. [d]n is dimensionless parameter. [e]R_{ct} is electron transfer resistance.

Table S3 CO_2 reduction performance of Zn-MOF/CP cathode in various electrolytes at -2.2 V vs Ag/Ag^+ after 2 hrs.[a]

Entry	Electrolytes	j_{tot} [mA·cm^{-2}]	FE_{CH_4} [%]	FE_{CO} [%]	FE_{HCOOH} [%]	FE_{H_2} [%]
1	0.01M $TBABF_4$/DMF	1.0±0.06	26.3	-	6.8	59.6
2	0.1M $TBAPF_6$/MeCN	3.1±0.5	23.2	15.4	19.0	48.6
3	0.1M $BmimBF_4$/MeCN	5.5±0.9	10.3	8.3	56.4	29.0

[a]The Zn-MOF/CP cathode was prepared using Zn-MOF synthesized at x=0.38 for reduction of CO_2.

Table S4 The overpotentials at -2.2 V vs Ag/Ag^+, linear range in Tafel plots, and Tafel slopes for the main product of different kinds of cathodes.

entry	Cathode	Main prouduct	Linear range [V]	Tafel slope [mV·dec^{-1}]
1	Zn-MOF/CP[a]	CH_4	0.19-0.37	146
2	Au	CO	0.05-0.27	186
3	Ag	CO	0.23-0.38	152
4	Pt	H_2	0.3-0.54	197
5	Fe	CH_4	0.38-0.6	253
6	Zn	CH_4	0.2-0.43	209
7	Cu	CH_4	0.45-0.65	200

[a]The cathode was prepared using Zn-MOF synthesized at x=0.38.

Table S5 CH_4 selectivity using various cathodes reported in literature.

entry	Electrode	Electrolytes	Potential [V]	FE_{CH_4} [%]	Ref.
1	Cu/C (7 nm)	0.1 M $NaHCO_3$	-1.35V vs Ag/AgCl	76	S7
2	Cu (210)	0.1 M $KHCO_3$	-1.52V vs Ag/AgCl	60.5	S8
3	Cu	0.1 M $KHCO_3$	-1.05V vs RHE	24.4	S9
4	Cu	0.1 M $KHCO_3$	-2.2V vs SCE	60	S10
5	Ag	0.1 M $KHCO_3$	-1.35±0.025 vs RHE	0.07	S11
6	Zn	0.1 M $KHCO_3$	-1.31±0.009 vs RHE	0.16	S11
7	Cu	0.1 M $KHCO_3$	-1.05±0.008 vs RHE	24.4	S11
8	Ni	0.1 M $KHCO_3$	-1.04±0.018 vs RHE	0.4	S11
9	Pt	0.1 M $KHCO_3$	-0.75±0.006 vs RHE	0.02	S11
10	Fe	0.1 M $KHCO_3$	-0.54±0.006 vs RHE	0.01	S11
11	Polycrystalline Cu	0.1 M $KHCO_3$	-1.0V vs RHE	43	S12
12	Annealed Cu	0.1 M $KHCO_3$	-1.0V vs RHE	22	S12
13	Cu_2O (200 nm)	0.1 M $KHCO_3$	-0.99V vs RHE	9.85	S13
14	Cu (210)	0.1 M $KHCO_3$	-1.52V vs SHE	64.0	S14
15	Cu (755)	0.1 M $KHCO_3$	-1.43V vs SHE	62.9	S14
16	Cu nanoparticles (1.9 nm)	0.1 M $KHCO_3$	-1.1V vs RHE	15	S15

.

References

S1. Q. B. Liu, M. H. A. Janssen, F. van Rantwijk and R. A. Sheldon, *Green Chem.*, 2005, **7**, 39.

S2. W. T. Shang, X. C. Kang, H. Ning, J. L. Zhang, X. G. Zhang, Z. H. Wu, G. Mo, X. Q. Xing and B. X. Han, *Langmuir*, 2013, **29**, 13168.

S3. S. C. Canobre, D. A. L. Almeida, C. P. Fonseca, S. Neves, *Electrochim. Acta* 2009, *54*, 6383-6388; b) S. A. Mamuru, K. I. Ozoemena, T. Fukuda, N. Kobayashi and T. Nyokong, *Electrochim. Acta*, 2010, **55**, 6367.

S4. P. Acevedo-Peña, M. Haro, M. E. Rincón, J. Bisquert and G. Garcia-Belmonte, *J. Power Sources*, 2014, **268**, 397.

S5. V. V. Pavlishchuk, A. W. Addison, *Inorg. Chim. Acta*, 2000, **298**, 97.

S6. B. A. Rosen, A. Salehi-Khojin, M. R. Thorson, W. Zhu, D. T. Whipple, P. J. A. Kenis and R. I. Masel, *Science*, 2011, **334**, 643.

S7. K. Manthiram, B. J. Beberwyck and A. P. Aivisatos, *J. Am. Chem. Soc.*, 2014, **136**, 13319.

S8. Y. Hori, I. Takahashi, O. Koga and N. Hoshi, *J. Phys. Chem. B*, 2002, **106**, 15.

S9. K. P. Kuhl, E. R. Cave, D. N. Abram and T. F. Jaramillo, *Energy Environ. Sci.*, 2012, **5**, 7050.

S10. Y. Momose, K. Sato and O. Ohno, *Surf. Interface Anal.*, 2002, **34**, 615.

S11. K. P. Kuhl, T. Hatsukade, E. R. Cave, D. N. Abram, J. Kibsgaard and T. F. Jaramillo, *J. Am. Chem. Soc.*, 2014, **136**, 14107.

S12. C. W. Li and M. W. Kanan, *J. Am. Chem. Soc.*, 2012, **134**, 7231.

S13. D. Ren, Y. L. Deng, A. D. Handoko, C. S. Chen, S. Malkhandi and B. S. Yeo, *Acs Catal.*, 2015, **5**, 2814.

S14. *J. Mol. Catal. A-Chem.* 199, 39-47 (2003). Y. Hori, I. Takahashi, O. Koga and N. Hoshi, *J. Mol. Catal. A-Chem.*, 2003, **199**, 39.

S15. R. Reske, H. Mistry, F. Behafarid, B. R. Cuenya and P. Strasser, *J. Am. Chem. Soc.*, 2014, **136**, 6978.
